# A Glance into MTHFR Deficiency at a Molecular Level

**DOI:** 10.3390/ijms23010167

**Published:** 2021-12-23

**Authors:** Castrense Savojardo, Giulia Babbi, Davide Baldazzi, Pier Luigi Martelli, Rita Casadio

**Affiliations:** 1 Biocomputing Group, Department of Pharmacy and Biotechnology, University of Bologna, 40126 Bologna, Italy; castrense.savojardo2@unibo.it (C.S.); giulia.babbi3@unibo.it (G.B.); davide.baldazzi8@unibo.it (D.B.); rita.casadio@unibo.it (R.C.); 2 Institute of Biomembranes, Bioenergetics and Molecular Biotechnologies (IBIOM), Italian National Research Council (CNR), 70126 Bari, Italy

**Keywords:** MTHFR deficiency, MTHFR variants, functional annotation, structural annotation, disease related variations, solvent accessibility, ΔΔG predictions, consensus method, protein-protein interactions, disease HMM models

## Abstract

MTHFR deficiency still deserves an investigation to associate the phenotype to protein structure variations. To this aim, considering the MTHFR wild type protein structure, with a catalytic and a regulatory domain and taking advantage of state-of-the-art computational tools, we explore the properties of 72 missense variations known to be disease associated. By computing the thermodynamic ΔΔG change according to a consensus method that we recently introduced, we find that 61% of the disease-related variations destabilize the protein, are present both in the catalytic and regulatory domain and correspond to known biochemical deficiencies. The propensity of solvent accessible residues to be involved in protein-protein interaction sites indicates that most of the interacting residues are located in the regulatory domain, and that only three of them, located at the interface of the functional protein homodimer, are both disease-related and destabilizing. Finally, we compute the protein architecture with Hidden Markov Models, one from Pfam for the catalytic domain and the second computed in house for the regulatory domain. We show that patterns of disease-associated, physicochemical variation types, both in the catalytic and regulatory domains, are unique for the MTHFR deficiency when mapped into the protein architecture.

## 1. Introduction

The one-carbon metabolism cycle, including the folate and methionine cycles, is a critical pathway for cell survival. The human enzyme methylenetetrahydrofolate reductase (encoded by the gene MTHFR, UniProt code: P42898)) exchanges one-carbon unit from the folate to methionine cycle. This is exclusively used for methionine and S-adenosylmethionine (SAM) synthesis, and MTHFR is the rate-limiting enzyme in the methyl cycle, undergoing allosteric inhibition by its end product SAM (S-Adenosil-Methionine) [1,2,3,4]. The protein is functional in its homodimeric form [5].

MTHFR catalyzes the conversion of 5,10-methylenetetrahydrofolate to 5-methyltetrahydrofolate, a co-substrate for homocysteine re-methylation to methionine (EC number: 1.5.1.20). The recent release of its structure (PDB code: 6FCX, 0.25 nm resolution) highlights the organization of the protein into two flexible domains, one catalytic and one regulatory, with a connecting linker allowing domain-domain interactions, possibly due to a phosphorylation cascade. The structure clarifies the molecular mechanism of the reaction, which requires FAD as a cofactor, NAD(P)H to provide reducing equivalents and homodimerisation for allosteric regulation upon SAM binding at the regulatory domain [6]. The 36-residue N-terminal portion is not resolved in the available PDB file and MobiDB predicts only here a flexible region (https://mobidb.bio.unipd.it/P42898 accessed on 10 October 2021). The PDB contains the homodimeric protein organization.

MTHFR deficiency and upregulation result in various disease states, which have been extensively described in relation to a number of variants characterized in many studies. 109 MTHFR mutations have been reported in 171 families, including 70 missense mutations, 17 that primarily affect splicing, 11 nonsense mutations, seven small deletions, two no-stop mutations, one small duplication, and one large duplication [7]. Two other variants, A222V and E429A, distributed worldwide in the population, are characterized by a reduced enzymatic activity, and are associated to different risk factors [8,9]. Variations, reducing the MTHFR activity to different extents, result in hyperhomocysteinemia and varying severities of disease, including ischemic stroke, folate sensitive neural tube defects and schizophrenia [1]. Evidently, the protein is also an attractive drug target [10]. All known missense variations are distributed in the three-dimensionally resolved catalytic and regulatory domains.

In this study, we are interested in exploiting with computational tools the structural properties of the protein missense variations associated to the disease to highlight possible mechanisms of protein destabilization due to residue change. To this aim we first map disease variations on the protein structure in relation to their solvent accessibility and compute for the accessible variations their likelihood of being involved in protein-protein interactions. We also compute the Gibbs free energy change (ΔΔG) for each variation with a consensus method and find that positions 387 (G387D), 506 (Y506D) and 628 (L628T) of the protein homodimeric interface at the level of the two regulatory domains, besides being correctly predicted as interaction sites, are also destabilizing the protein homodimer. This corroborates the relevance of the interaction of the two regulatory domains for the stability of the functional protein. We then grouped all the disease-related variations according to their physicochemical types and mapped them into the computed HMM modelled architecture of the protein. By this we establish a link among protein domains and variation types, which is a unique marker of the MTHFR deficiency.

## 2. Results

Application of state-of-the art tools for functional annotation of a protein is common routine in the field of computational biology. Here, having the solved structure of the MTHRF gene, we aim at highlighting possible structural properties of the missense variations associated to the deficiency, and most cases associated to a decreased biochemical efficiency. Our goal is to relate computational properties, such as solvent exposure, being an interaction site and promote protein instability, to their annotation of being disease-associated. This highlights some interesting properties of the disease related variations and in turn benchmarks tools in the difficult task of their prediction.

### 2.1. MTHFR and Protein-Protein Interactions

Large scale experiments of interactomics indicate that human MTHFR interacts with many specific partners. BioGRID (https://thebiogrid.org/ accessed on 10 October 2021), the Database of Protein, Genetic and Chemical Interactions, lists 33 physical interactors, 22 of which are also present in IntAct (https://www.ebi.ac.uk/intact/ accessed on 10 October 2021), the other molecular data base collecting data from large scale experiments. It is worth noticing that none of the enzymes involved in the folate and methionine cycles are present among the physical interactors, and that many membrane and nuclear proteins are in the interacting protein pool. Why this is so perhaps deserves more experiments, and it can be interpreted considering the presence of MTHFR in different cell compartments, including its putative interaction with mitochondria, endoplasmic reticulum, and the nucleus [1]. For the time being, we can compute the likelihood of solvent-exposed residues to be in contact with a putative partner. We adopt our ISPRED4 predictor [11], which is based on machine learning, and it is specifically suited to compute the likelihood of an exposed residue to be involved in a protein contact. We compute 44 interacting sites in the protein structure (see Supplementary Material Table S1 for details), 17 of which are at the interface between the two regulatory domains of the homodimer. Many of the interactions reported in the databases are likely to be non-obligate and therefore different interactions can involve the same sites, in different compartments and phases of the cell lifespan. This can be considered the reason why the number of interaction sites as derived from a structure rarely coincides with the number of interactors as derived from large scale experiments. In the following, our interest is on disease-related variations which are in interaction sites and whose functional annotations are already documented (Table 1).

**Figure 1:**
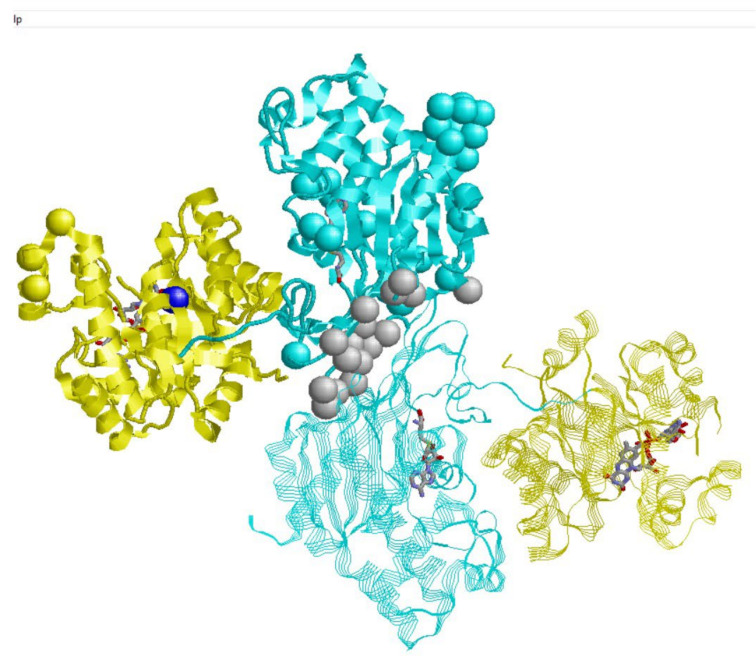
Protein-protein interaction sites predicted with ISPRED4 [10] on the MTHFR PDB 6FXC. The catalytic and regulatory domains are depicted in yellow and pale blue, respectively. Interaction sites are represented with hard spheres centered on the C-alpha atom of the specific residue. Grey spheres are residues in the homodimeric interface, correctly predicted as interaction sites.

In Figure 1, predicted contacts are represented with hard spheres centered on the C-alpha atom of the specific residue. The color code follows the organization of the protein in the catalytic (yellow) and regulatory (pale blue) domain, inclusive of the linker region [6]. The bound FAD and SAH molecules, present in the protein crystal (6FCX), are also shown for clarity, and their binding sites are relevant for protein catalytic activity.

Interestingly enough, we correctly predict the interface region of the homodimer (hard spheres in grey). Other predicted PPI sites are distributed in different regions of the protein surface. These residues are candidates for taking part in the interaction with the 33 proteins reported in the IntAct and BioGRID databases. Only in position 387 (G387D), 506 (Y506D) and 628 (L628T) of the homodimeric interface at the level of the regulatory domain do the predicted interaction sites coincide with missense variations associated with the MTHFR disease. These variations are also predicted as destabilizing (see below). This observation finally highlights the role of the regulatory domain interactions not only in being part of the protein functional stability, but also in playing a role in the disease [5].

### 2.2. MTHFR and Protein Stability

We can investigate whether disease-related missense variations are related to protein instability. To this aim, we adopt a consensus method, computing (with three state-of-the- art methods) the Gibbs free energy change (ΔΔG) associated with a specific variation in the protein. We select a consensus method, given the variability of the different methods in predicting the ΔΔG values [12], and adopt three of the art methods: INPS-MD [13] is based on machine learning, FoldX [14] on statistical potentials, and PoPMuSiC2 [15] on statistical potentials and machine learning.

**Table 1:** MDHFR deficiency-related variations.

		ΔΔG (kcal/mol)				
Variation	Effects	INPS3D	FoldX	PoPMuSiC2	ISPRED4	RSA (%)
**Catalytic Domain**						
R46Q	No effect on NAD(P) affinity	−0.76	−0.26	−1.05	N	29
R46W	No effect on NAD(P) affinity	−0.5	−1.04	−0.35	N	29
**R51P**		**−1.24**	**−1.13**	**−1.47**	**N**	**49**
R52Q	Reduced affinity for NAD(P)	−1.06	0.08	−0.77	N	23
**W59C**		**−1.59**	**−3.58**	**−2.52**	**N**	**2**
**W59S**		**−2.67**	**−3.92**	**−3.3**	**N**	**2**
P66L	NAD(P) binding site	−0.46	−4.09	−0.01	N	20
R68G	Reduced affinity for NAD(P) NAD(P) binding site	−0.92	−0.40	−0.59	N	96
R82W	No effect on NAD(P) affinity	−0.66	0.2	−0.81	N	44
**A113T**	**No effect NADPH**	**−1.17**	**−1.44**	**−1.71**	**N**	**0**
**A116T**		**−0.65**	**−2.29**	**−1.95**	**N**	**0**
H127Y	FAD binding site	−0.18	1.37	−0.33	N	5
**T129N**	**Reduced affinity for NAD(P)** **FAD binding site**	**−1.17**	**−1.37**	**−0.81**	**N**	**7**
**C130R**	**No effect on NAD(P) affinity**	**−1.99**	**−16.08**	**−1.34**	**N**	**1**
T139M		−0.34	0.83	0.29	N	18
Q147P		−0.46	−2.95	−0.91	N	73
**G149V**		**−1.02**	**−13.0**	**−3.26**	**N**	**2**
**I153M**	**No effect on NAD(P) affinity**	**−1.56**	**0.18**	**−1.71**	**N**	**1**
R157Q	No effect on NAD(P) affinity FAD binding site	−1.31	−0.72	−0.57	N	25
A175T	Reduced affinity for NAD(P) FAD binding site	−1.13	−0.73	−0.54	N	8
**H181D**		**−1.79**	**−2.23**	**−1.5**	**N**	**10**
**R183Q**	**No effect on NAD(P) affinity**	**−1.48**	**−3.34**	**−0.82**	**N**	**16**
**C193Y**		**−1.19**	**−10.91**	**−0.03**	**N**	**17**
A195V	Reduced affinity for NAD(P) FAD binding site	−0.43	0.39	0	N	11
**G196D**	**Reduced affinity for NAD(P)**	**−1.08**	**−3.26**	**−1.18**	**N**	**2**
P202T	FAD binding site	−0.73	−1.57	−0.16	N	68
V218L	Decreased affinity for FAD	−1.00	−0.42	−0.42	N	12
A222V *	Decreased affinity for FAD	−0.71	−1.08	−0.09	N	11
**I225L**	**No effect on NAD(P) affinity**	**−1.32**	**−0.57**	**−1.17**	**N**	**0**
**T227M**		**−1.58**	**−2.9**	**−0.14**	**N**	**1**
P251L		−0.56	0.62	−0.68	N	38
**V253F**	**Reduced affinity for NAD(P)**	**−0.82**	**−1.26**	**−1**	**N**	**0**
**P254S**	**No effect on NAD(P) affinity**	**−1.22**	**−3.7**	**−0.86**	**N**	**0**
G255V		−0.55	−2.81	0.33	N	1
**I256N**		**−3.24**	**−3.27**	**−2.47**	**N**	**1**
**F257V**		**−1.34**	**−1.61**	**−1.83**	**N**	**11**
**L323P**	**Substrate binding site** **NAD(P) binding site**	**−2.19**	**−4.95**	**−1.94**	**N**	**32**
**N324S**		**−0.77**	**−3.52**	**−1.92**	**N**	**8**
R325C	Substrate binding site	−0.78	0.41	−0.34	N	43
**L333P**		**−3.39**	**−6.05**	**−3.62**	**N**	**0**
R335C		−0.67	−1.14	−0.86	N	60
**Regulatory Domain**						
**M338T**		**−1.58**	**−3.74**	**−1.21**	**N**	**18**
**W339G**		**−2.78**	**−4.46**	**−2.55**	**N**	**20**
R345C		−0.67	−1.31	−0.23	N	43
**P348S**	**Reduced affinity for NAD(P)** **SAH binding site**	**−1.19**	**−3.53**	**−1.16**	**N**	**26**
H354Y	Reduced affinity for NAD(P)	−0.24	−0.2	−0.67	N	18
**R357C**		**−1.32**	**−2.3**	**−1.54**	**N**	**5**
**R357H**		**−1.28**	**−1.09**	**−0.29**	**N**	**5**
**R363H**	**Reduced affinity for NAD(P)**	**−1.39**	**−1.34**	**−0.83**	**N**	**6**
K372E	Reduced affinity for NAD(P)	−0.46	0.99	−0.31	N	52
**R377C**	**Reduced affinity for NAD(P)**	**−1.17**	**−3.99**	**−1.4**	**N**	**0**
**R377H**	**Reduced affinity for NAD(P)**	**−1.2**	**−4.59**	**−0.68**	**N**	**0**
**W381R**		**−1.83**	**−2.29**	**−1.95**	**N**	**14**
**G387D**	**Reduced affinity for NAD(P)**	**−0.82**	**−3.35**	**−1.31**	**I**	**33**
G390D		−0.88	−2.23	0.13	N	64
**W421S**	**Reduced affinity for NAD(P)**	**−3.07**	**−6.97**	**−4**	**N**	**1**
E429A *		−0.13	−0.79	0.2	N	50
**F435S**		**−3.45**	**−5.56**	**−2.94**	**N**	**1**
S440L		0.03	2.15	−0.55	N	25
**Y506D**	**Reduced affinity for NAD(P)**	**−1.77**	**−5.1**	**−3.16**	**I**	**61**
**Y512C**		**−1.94**	**−4.31**	**−2.18**	**N**	**2**
R535Q		−0.79	−1.61	−0.77	N	25
R535W		0.07	−1.39	−0.26	N	25
**V536F**	**Reduced affinity for NAD(P)**	**−1.38**	**−3.21**	**−0.6**	**N**	**1**
P572L	Reduced affinity for NAD(P)	−0.43	−7.32	−0.09	N	0
**V574G**	**Reduced affinity for NAD(P)**	**−3.32**	**−4.13**	**−3.47**	**N**	**1**
**V575G**	**Reduced affinity for NAD(P)**	**−3.64**	**−4.06**	**−3.07**	**N**	**8**
E586K		−0.8	−5.23	−0.99	N	1
**L598P**	**Reduced affinity for NAD(P)**	**−2.49**	**−7.34**	**−2.68**	**N**	**22**
**S603C**		**−1.03**	**−1.81**	**−0.7**	**N**	**15**
**L628P**	**Reduced affinity for NAD(P)**	**−0.83**	**−4.47**	**−2.25**	**I**	**57**
**M338T**		**−1.58**	**−3.74**	**−1.21**	**N**	**18**

The table lists 72 variations associated to the MTHFR deficiency, as reported in the MTHRF UniProt file MTHFR, UniProt code: P42898 and in [7]. * Variations are described in [8] (A222V) and [9] (E229), respectively. Effects of the variations on the MTHFR enzymatic activity are listed when reported. Bold style indicates variations for which at least two of the three methods adopted for computing ΔΔG (INPS3D, [13]; FoldX [14]; and PoPMuSiC2 [15], compute negative results, lower than −1 kcal/mol, indicating protein destabilization (for details see text). For completeness, we include results (I, Interaction; N, No Interaction) of the Interaction site prediction method (ISPRED4) [11] and values of the relative solvent accessibility (RSA%) (see Materials and Methods for details) (second to last column and right-most column, respectively).

We select as a threshold value |1 kcal/mol|, which takes into account the variability of the experimental thermodynamic data on protein stability adopted for training the predictors. In Table 1, we list the 42 disease-related variations in the catalytic domain and the 30 disease related variations in the regulatory domain. Alongside this, we indicate the corresponding effects on the protein function, the computed ΔΔG according to the three predictors, the prediction of the wild-type residue to be in contact or not and the computed relative solvent accessibility [16]. It appears that 22 variations in the catalytic domain and 20 variations in the regulatory domain decrease protein stability, according to at least two of the three predictors. Among the remaining ones, seven are in the NAD(P)H binding site, and the other seven are in the FAD binding site, respectively. These variations, as reported in Table 1, decrease the binding affinity without perturbing the protein stability, including A222V and E429A. In any case, the available structure 6FCX contains an A in position 429 instead of E, and in this case, we compute ΔΔG of the reverse variation [12]. R325C, in the substrate-binding site, decreases substrate affinity without affecting protein stability.

In the protein catalytic domain, we map 41 disease related variations, 14 of which are exposed and not in interaction sites, 20 are destabilizing and mostly (90%) buried. In the protein regulatory domain, we map the remaining 31 disease related variations, 12 of which are exposed, 21 are destabilizing and 15 of these are buried. Interestingly, positions 387 (G387D), 506 (Y506D) and 628 (L628T) at the protein homodimeric interface, correctly predicted as interaction sites (see above), promote also protein destabilization, supporting a role of the regulatory domain interactions in the stability of the functional protein homodimeric complex. Overall, 61% of the disease related variations are affecting the protein stability and most of them have been experimentally found to promote instability of cofactor binding.

Out of the pool of the MDRFH deficiency variations listed in Table 1, UniProt in the protein file P42898 lists eight other variations with a dbSNP code (https://www.ncbi.nlm.nih.gov/snp/ accessed on 10 October 2021) not yet associated to disease (likely benign?). Six of these maps into the protein structure and two of them, (G422R, exposed, and G566E, at the homodimer interface) destabilize the protein structure according to our criterion. Results are in line with previous observations highlighting how protein instability is not a necessary condition for being disease-related [17,18], although in this specific case many of the variations are indeed destabilizing the protein organization (Table 1).

### 2.3. MTHFR Deficiency and Its Structural Model

**Figure 2:**
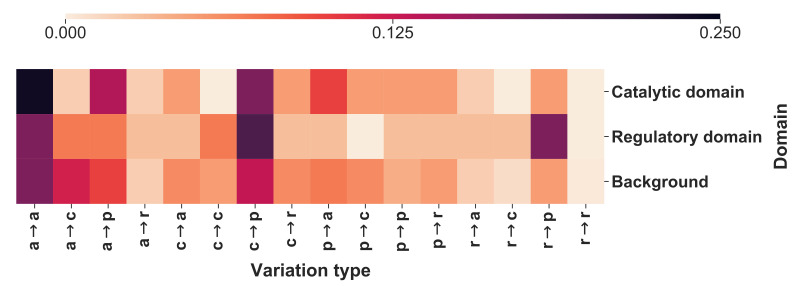
The heatmap reporting the frequency of each variation type as observed within the catalytic and the regulatory domains. The background distribution has been computed considering 22,763 pathogenic variations from Humsavar in 2513 proteins. In variation types, labels are as follows: a, apolar; c, charged; p, polar; and r, aromatic (for details see text). Differences between Catalytic and Regulatory sites are significant at 10% when a Chi-square test is applied after adding pseudocounts (with value 0.5) for regularization.

Recently we introduced the concept of mapping disease variation types into associated Pfam structural protein models (https://pfam.xfam.org accessed on 10 October 2021), finding that by this it is possible to establish a relation among genes and maladies [18,19]. Indeed, mapping of variation types into Pfam is unique for a given disease. Here we exploit our strategy with MTHFR, considering Pfam 02219 for the catalytic domain. This Pfam is shared by similar proteins in Eukarya, Bacteria and Archaea. The regulatory domain does not have a Pfam model and is present only in Eukarya. We build a model for the regulatory domain aiming for a structural representation of the complete protein. The model is an HMM of the profile of a multiple alignment of some 50 sequences from Eukarya with a length similar to that of MTHFR. The model includes the linker, and it spans from residue 336 to residue 566 (see Supplementary Material, where the Pfam-like model of the regulatory domain is reported). We then converted the disease related variations of Table 1 into variation types (apolar (G, A, V, I, L, P, M); polar (S, T, C, N, Q, H); aromatic (F, W, Y); charged (D, E, K, R) giving rise to 16 possible variation types. We associated MTHFR related variation types to the protein architecture, as represented by P02219 and our Pfam-like model of the regulatory domain. The frequency of the variation types in each domain is represented in Figure 2.

It appears that the variational pattern is different in the two domains and different from the background variational pattern, obtained considering the pathogenic variations from Humsavar (https://www.uniprot.org/docs/humsavar accessed on 10 October 2021), in 2513 human proteins (22,763 disease related variations).

## 3. Materials and Methods

### 3.1. Characterization of Protein Surface and Annotation of Protein-Protein Interaction Sites

The solvent accessibility of residues of PDB entry 6FCX, chain A, [6] was computed with the DSSP program (https://swift.cmbi.umcn.nl/gv/dssp/DSSP_3.html accessed on 10 October 2021) and normalized with respect to the residue-specific maximal accessibility values as previously described [16]. Residues interacting in the homodimer interface are those undergoing a decrease of the absolute solvent accessibility (ASA) ≥ 1 Å^2^ in the complex with respect to the isolated monomer.

Protein-protein interaction sites were predicted with ISPRED4 [11], a tool based on support vector machines and grammatical restrained hidden conditional random fields that integrate 46 different features extracted from the monomer sequence, its multiple sequence alignment against the UniProt database and its 3D structure. ISPRED4 has been trained and cross-validated on 151 protein complexes and reaches a per-residue Matthews correlation coefficient of 0.48 and an overall accuracy of 0.85. Similar values are obtained on blind test sets, and therefore ISPRED4 is one of the top-performing tools for the computational annotation of protein-protein interaction sites.

### 3.2. Prediction of ∆∆G Changes upon Single Residue Variation

The possible effect on protein stability induced by single residue variation starting from protein structure has been predicted with three state-of-the-art methods: (i) INPS3D [13], a tool based on a machine-learning approach; (ii) FoldX [14], that estimated energy changes on the basis of a knowledge-based potential; and (iii) PoPMuSic2 [15], a method implementing a combination of statistical potentials optimized with a neural network. The following convention has been adopted for the definition of the ∆∆G sign:

∆∆G = (∆G_wt_ − ∆G_mut_) (1)

where ∆G_wt_ and ∆G_mut_ are the folding free energy of the wild-type and mutated proteins, respectively. Negative values of ∆∆G mean that the mutated form is less stable than the wild-type. We considered as destabilizing the variations for which at least two methods predict ∆∆G ≤ −1 kcal/mol.

Since the structure 6FCX carries the mutant allele (A) in position 429, the thermodynamic effect of variation E429A was estimated by computing the ∆∆G of variation A429E on the crystal and applying the antisymmetric principle.

### 3.3. Pfam-Like Model of the Regulatory Domain

From the UniRef50 cluster UniRef50_P42898 (https://www.uniprot.org/uniref/UniRef50_P42898 accessed on 10 October 2021), we collected 150 complete protein sequences from Eukarya and with length ranging between 640 and 670 residues. These sequences cover both domains of human MTHFR protein and share more than 50% sequence identity with it. We aligned the sequences with ClustalOmega (https://www.ebi.ac.uk/Tools/msa/clustalo/ accessed on 10 October 2021) and extracted the multiple sequence alignment of the regulatory domain, spanning from position 336 to 566 in the human sequence. We then trained a HMM, with HMMER 3.3.2 (http://hmmer.org/ accessed on 10 October 2021). The trained model is available in the Supplementary Materials.

## 4. Conclusions

In this paper we exploit different computational methods for refining the annotation of the disease related variants of MTHFR, promoting MTHFR deficiency. Due to the numerous biological processes in which the protein is directly and/or indirectly involved, MTHFR is of particular interest, since its partial or total disfunction may have a range of effects on human health, spanning from mild to lethal ones. Among the known 72 disease related variations, we characterize those that are at the protein surface, participate into protein-protein contacts and are at the homodimer interface which involves the protein regulatory domain. We also highlight other properties of the protein, like the exposed residues that eventually participate in the protein-protein interaction (Table S1). Furthermore, we show that 61% of the disease related variants are destabilizing the protein, highlighting a possible source of structural destabilization causing the decreased binding affinity of the protein cofactors when documented. Noteworthy is that positions 387 (G387D), 506 (Y506D), and 628 (L628T) in the interface of the two regulatory domains of the homodimeric protein, besides being disease associated, are correctly predicted as interaction sites, and predicted also as destabilizing. This confirms the role of the regulatory domains interaction in supporting the homodimeric functional unit [5].

Finally, we propose a structural variational model for MTHFR deficiency by associating variation types to the protein architecture, as modelled with HMMs representing the catalytic and regulatory domain, respectively.

## Data Availability

The data presented in this study are available in the tables, figures and Supplementary Materials of this article.

## Supplementary Materials

The following supplementary files are available online at https://www.mdpi.com/article/10.3390/ijms23010167/s1.
