# Supplementary material for: A Glance into MTHFR Deficiency at a Molecular Level
Source: Int J Mol Sci. 2021 Dec 23;23(1):167. doi: 10.3390/ijms23010167 (PMC8745156; doi:10.3390/ijms23010167)
Supplement: Supplementary file 1 [file ijms-23-00167-s001.zip › Table S1.pdf]

Table S1. Protein-protein interaction sites predicted with ISPRED4 on the MTHFR PDB structure 6FCX, chain A. For each site we report the position, the residue, the relative solvent accessibility (RSA), the probability of being an interaction site as provided by ISPRED4 and whether the site is also reported in the crystallographic homodimeric interface or not.

Position	Residue	RSA (%)	ISPRED4 probability	Homodimeric interface
283	V	44	0.82	No
285	E	46	0.63	No
289	D	96	0.75	No
386	N	52	0.73	Yes
387	G	33	0.59	Yes
388	R	62	0.78	Yes
397	F	78	0.94	No
398	G	60	0.94	No
399	E	36	0.95	No
402	D	69	0.7	No
404	Y	88	0.9	No
405	L	59	0.95	No
406	F	77	0.61	No
465	S	62	0.53	No
466	L	47	0.94	No
489	N	58	0.57	Yes
502	P	29	0.67	No
506	Y	61	0.78	Yes
526	L	33	0.51	No
548	I	46	0.72	No
553	E	88	0.73	Yes
554	L	59	0.72	No
557	N	22	0.7	Yes
563	I	42	0.97	Yes
565	P	57	0.98	Yes
566	G	100	0.96	Yes
567	R	68	0.96	Yes
568	E	92	0.95	Yes
569	I	66	0.96	Yes
570	I	21	0.67	Yes
571	Q	21	0.51	Yes
578	V	57	0.93	No
626	F	25	0.94	Yes
628	L	57	0.68	Yes
642	L	46	0.98	No
643	L	42	0.96	No
644	N	39	0.9	No
645	A	61	0.95	No
646	E	99	0.96	No
647	N	52	0.98	No
648	L	93	0.83	No
649	Y	100	0.89	No
650	F	42	0.97	No
651	Q	57	0.78	No
